# Supplementary material for: G4mer: An RNA language model for transcriptome-wide identification of G-quadruplexes and disease variants from population-scale genetic data
Source: Nat Commun. 2025 Nov 20;16:10221. doi: 10.1038/s41467-025-65020-7 (PMC12635080; doi:10.1038/s41467-025-65020-7)
Supplement: Supplementary file 1 — Supplementary Information [file 41467_2025_65020_MOESM1_ESM.pdf]

Supplementary Information

G4mer: An RNA language model for transcriptome-wide identification of G-quadruplexes and disease variants from population-scale genetic data

Farica Zhuang[1], Danielle Gutman[2], Nathaniel Islas[1], Bryan B. Guzman[3],
Alli Jimenez[4], San Jewell[2], Nicholas J. Hand[2,5],
Katherine Nathanson[6,7], Daniel Dominguez[3,4,8], Yoseph Barash[1,2*]

[1]Department of Computer and Information Science, University of Pennsylvania, Philadelphia, PA, USA.
[2]Department of Genetics, Perelman School of Medicine, University of Pennsylvania, Philadelphia, PA, USA.
[3]Department of Pharmacology, University of North Carolina at Chapel Hill, Chapel Hill, NC, USA.
[4]Department of Biochemistry and Biophysics, University of North Carolina at Chapel Hill, Chapel Hill, NC, USA.
[5]Institute for Translational Medicine and Therapeutics, Perelman School of Medicine, University of Pennsylvania, Philadelphia, PA, USA.
[6]Division of Human Genetics and Translational Medicine, Department of Medicine, Perelman School of Medicine, University of Pennsylvania, Philadelphia, PA, USA.
[7]Basser Center for BRCA, Abramson Cancer Center, Perelman School of Medicine, University of Pennsylvania, Philadelphia, PA, USA.
[8]RNA Discovery Center, The University of North Carolina at Chapel Hill, Chapel Hill, NC, USA.

*To whom correspondence should be addressed; E-mail: yosephb@upenn.edu

Supplementary Materials

Supplementary Figures

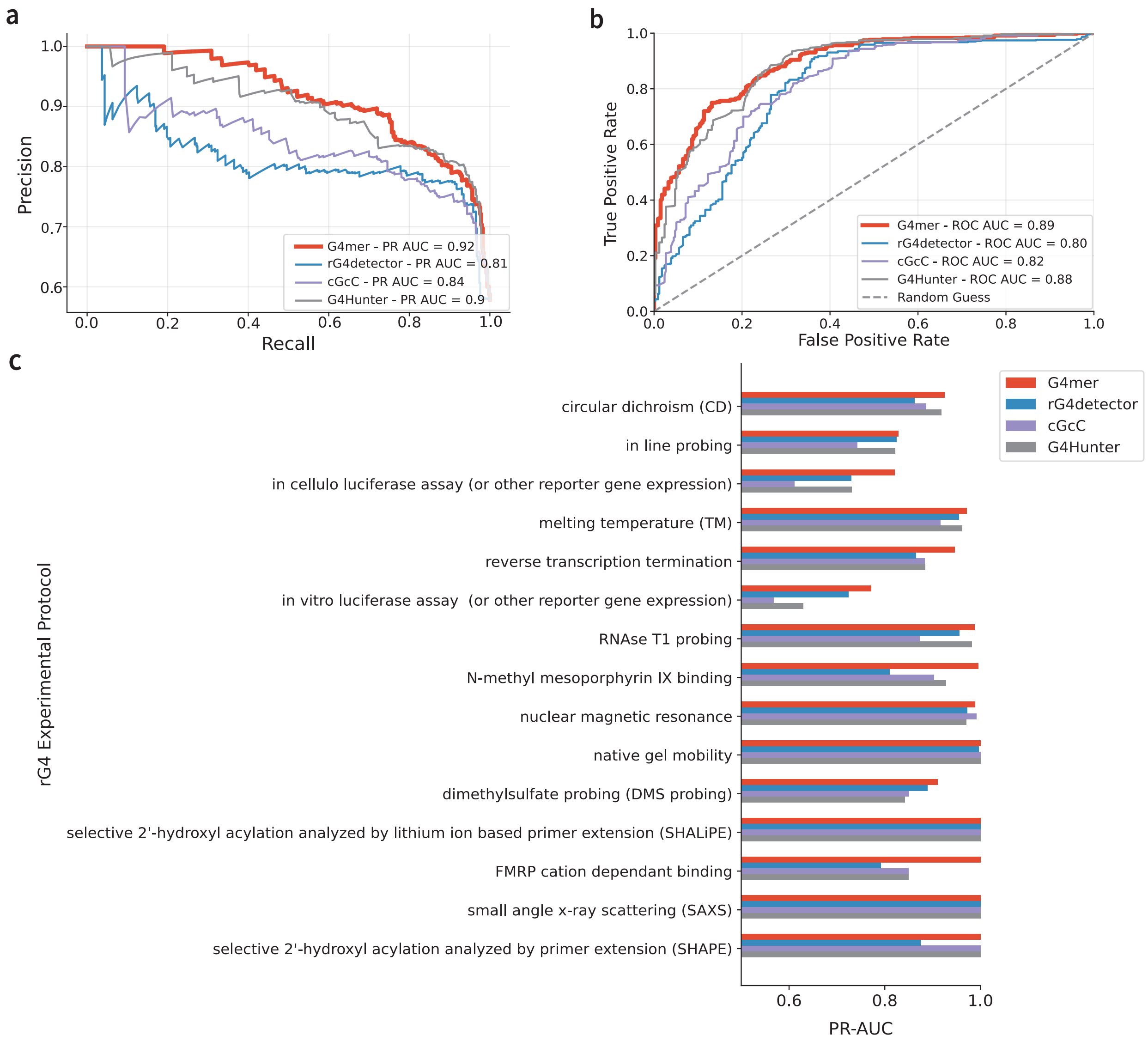

Supplementary Figure 1. Model performance comparison on sequences from G4RNA database validated using various experimental protocols. **(a)** ROC curves and **(b)** Precision-Recall (PR) curves comparing the performance of G4mer (red), rG4detector (blue), cGcC (purple), and G4Hunter (gray) in predicting rG4 formation for 795 sequences from G4RNA database. Sequences were validated by various experimental protocols. **(c)** PR-AUC comparison across the top 15 experimental protocols most frequently represented in the G4RNA database, ranked by number of sequences validated using each protocol. Protocols include a diverse set of experimental techniques including CD spectroscopy, SHAPE-based probing, and in cellulo luciferase assays (see Supplementary Table 4 for the exact number of sequences per protocol).

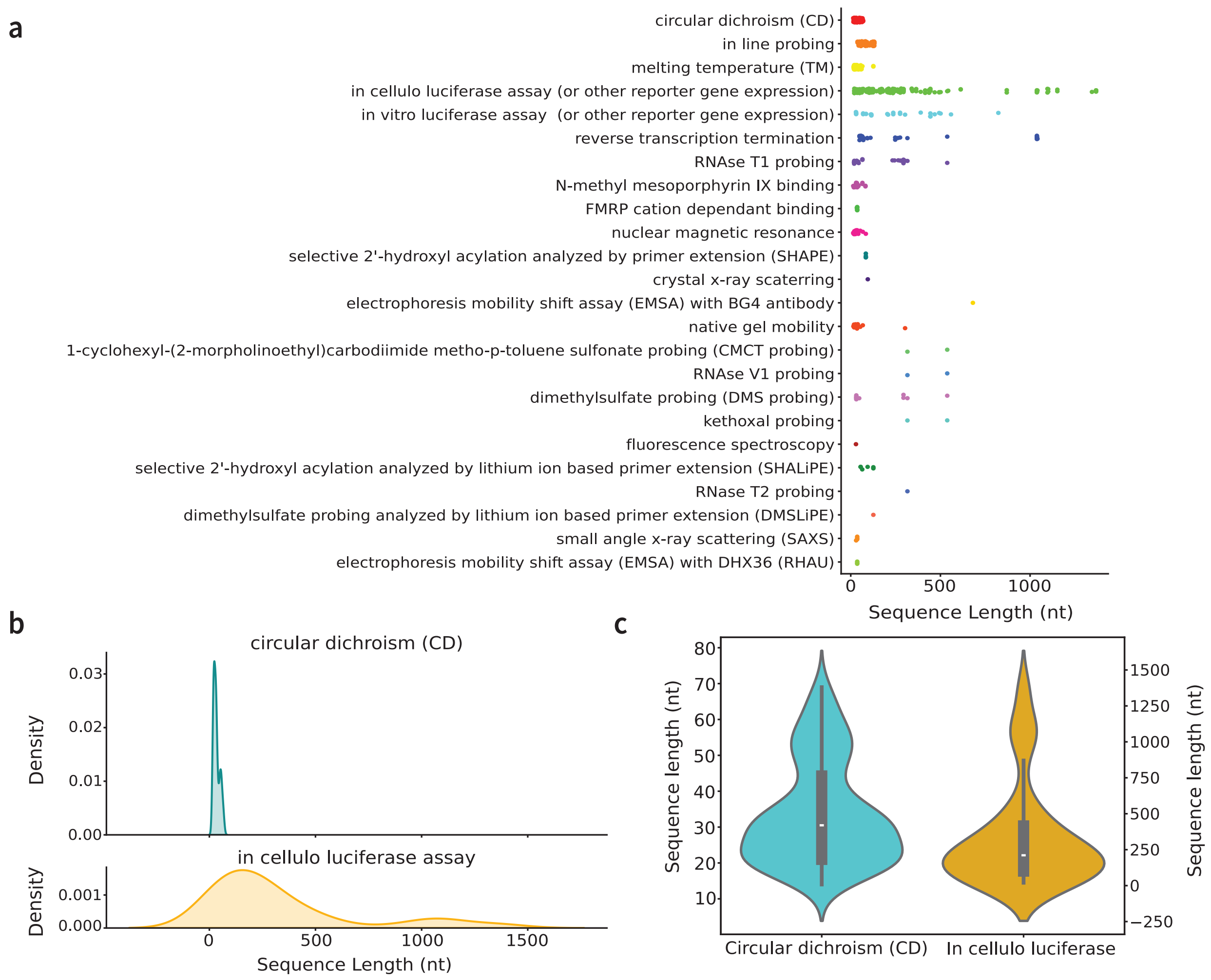

Supplementary Figure 2. G4RNA database sequence length distribution per experimental protocol. (a) Lengths of sequences tested by each of the 24 experimental protocols in G4RNA **(b)** Density plots showing the distribution of sequence lengths (in nucleotides) for two experimental protocols: circular dichroism (CD) and in cellulo luciferase assay. The top panel illustrates the density of sequence lengths for circular dichroism, where the majority of sequences are clustered in a narrow length range below 100 nt. The bottom panel shows the distribution for in cellulo luciferase assay, where the sequence lengths are more widely dispersed, covering a broader range of values, with the longest sequence being 1,368 nt. **(c)** Violin plots comparing the distribution of sequence lengths between circular dichroism (CD) and in cellulo luciferase assay. The left y-axis corresponds to the sequence lengths for CD, where the median is 30.5 not, as indicated by the horizontal white line within the violin plot. The right y-axis provides a scale for the in cellulo luciferase assay's broader sequence length range, where the median is 210 nt (see Supplementary Table 4 for the exact number of sequences per protocol).

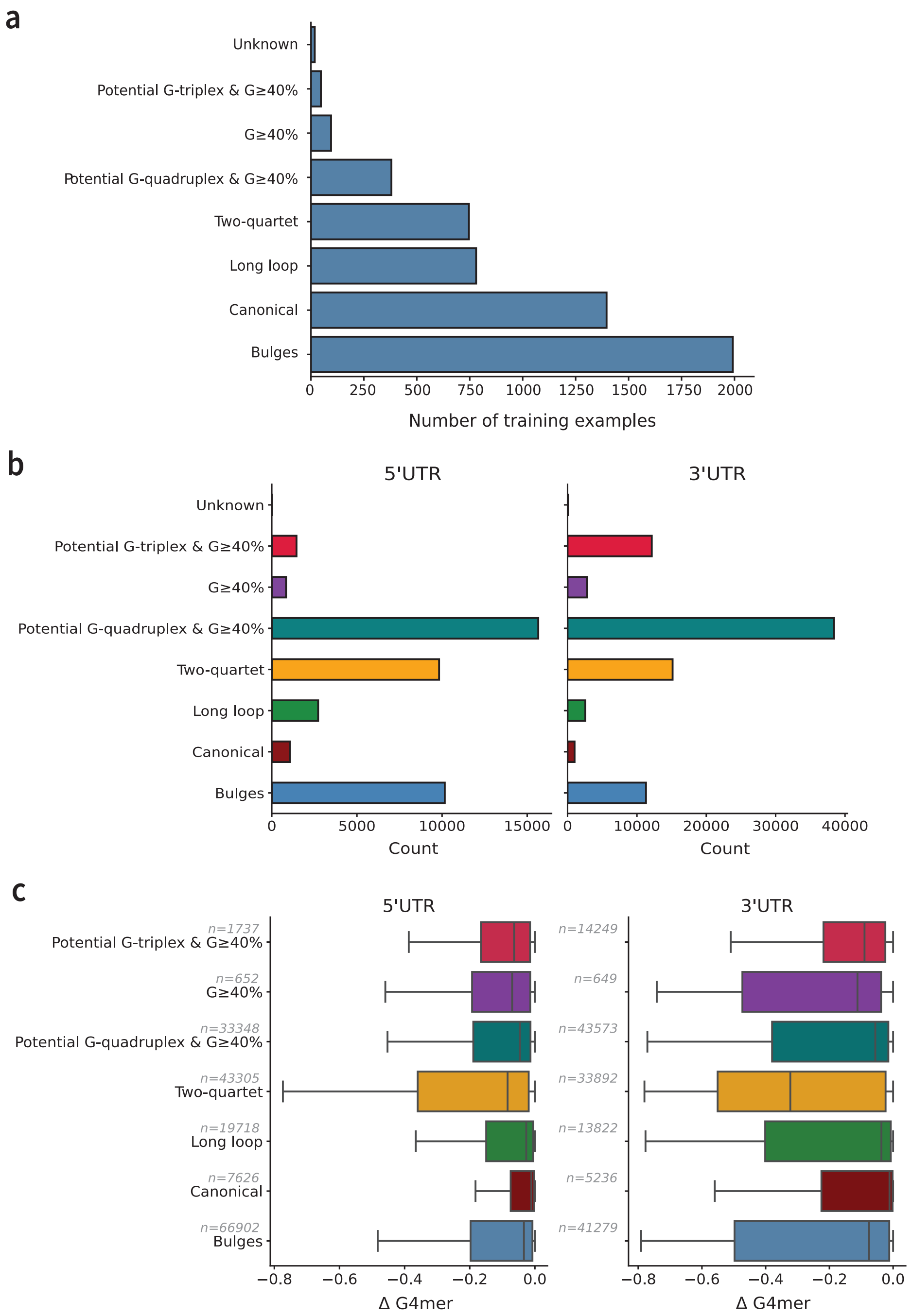

Supplementary Figure 3. Subtype-specific distributions and mutation effects across the transcriptome. **(a)** Distribution of rG4 subtypes in the G4mer training dataset. **(b)** Transcriptome-wide distribution of rG4 subtypes predicted by G4mer in 5′ and 3′ UTR regions, including the Unknown category. **(c)** Distribution of rG4-breaking effects (ΔG4mer) by subtype. Boxplots show the ΔG4mer distribution for all gnomAD variants that disrupt the predicted rG4 subtype. Counts (n) indicate the number of rG4-breaking variants analyzed per subtype in each UTR.

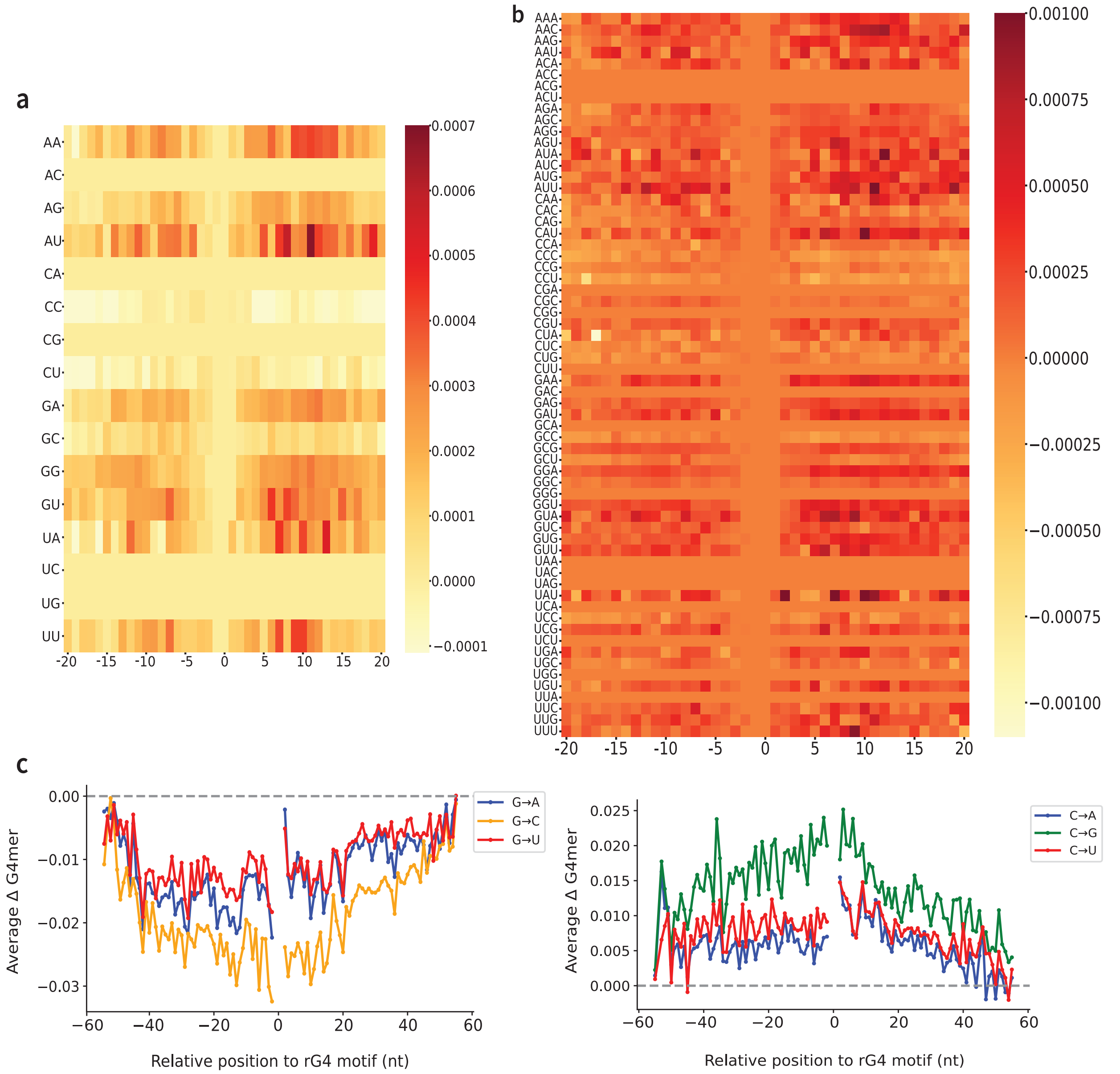

Supplementary Figure 4. EIG attribution and perturbation analyses of rG4 flanking regions. **(a, b)** Heatmaps of normalized attribution scores for **(a)** 2-mers and **(b)** 3-mers across positions relative to the G4 motif. Each row represents a unique k-mer, and each column corresponds to a position relative to the G4 motif. Color intensity reflects the magnitude of the attribution score, where higher attribution scores indicate a stronger contribution to rG4 structure prediction by G4mer. The baseline class consists of $n = 5{,}672$ non-rG4 sequences and the sample class (class of interest) consists of $n = 4{,}099$ UTR rG4 sequences. **(c)** Perturbation analysis of flanking nucleotides in $n = 6{,}707$ high-confidence rG4 sequences. The average change in G4mer prediction score (ΔG4mer) is shown for systematic substitutions of (left) guanines (G) and (right) cytosines (C) at each flanking nucleotide position relative to the rG4 motif.

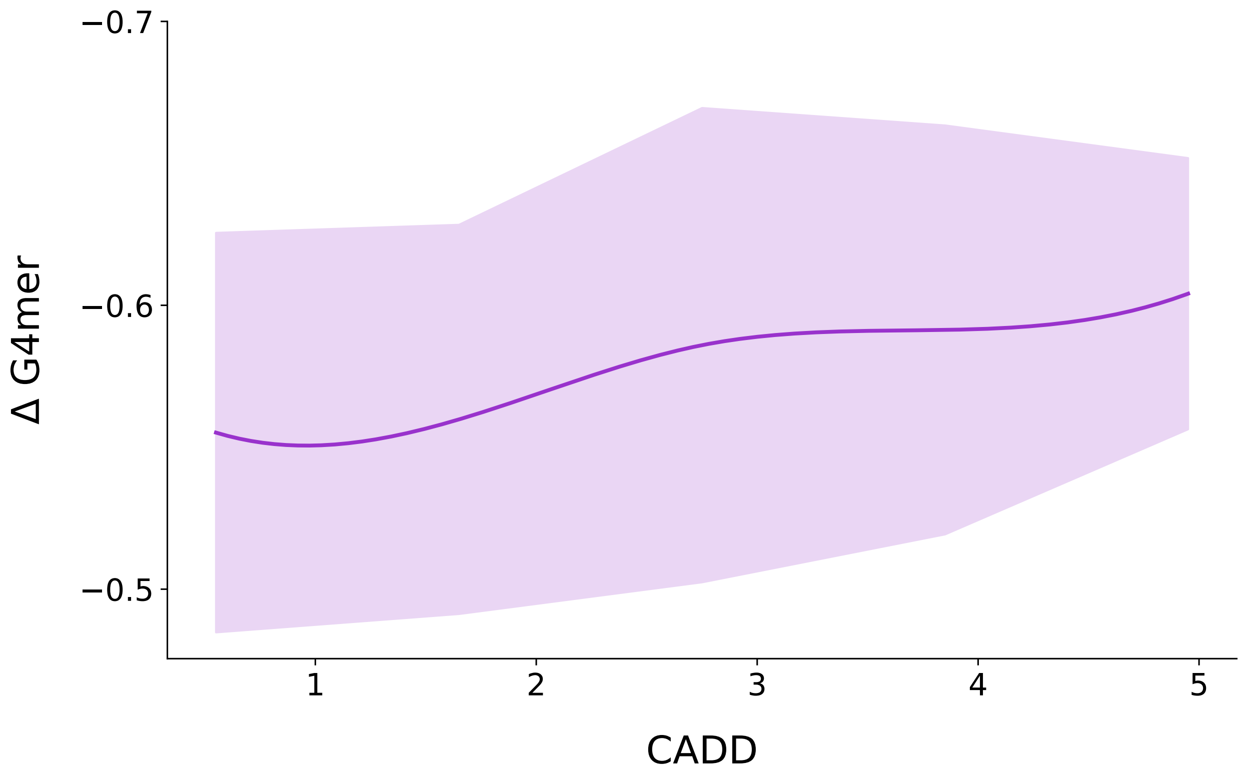

Supplementary Figure 5. Relationship between CADD scores and ΔG4mer values in the 3′ UTR. The plot shows the relationship between the Combined Annotation Dependent Depletion (CADD) scores and the change in G4mer prediction scores (ΔG4mer), based on $n = 6{,}599$ rG4-breaking variants in disease genes. The purple line represents the mean ΔG4mer value across different CADD scores, and the shaded area indicates the standard deviation. As the CADD score increases from 1 to 5, the ΔG4mer values show a subtle upward trend, suggesting a slight increase in deleteriousness of the variants that cause stronger rG4-breaking effects in the 3′ UTR regions.

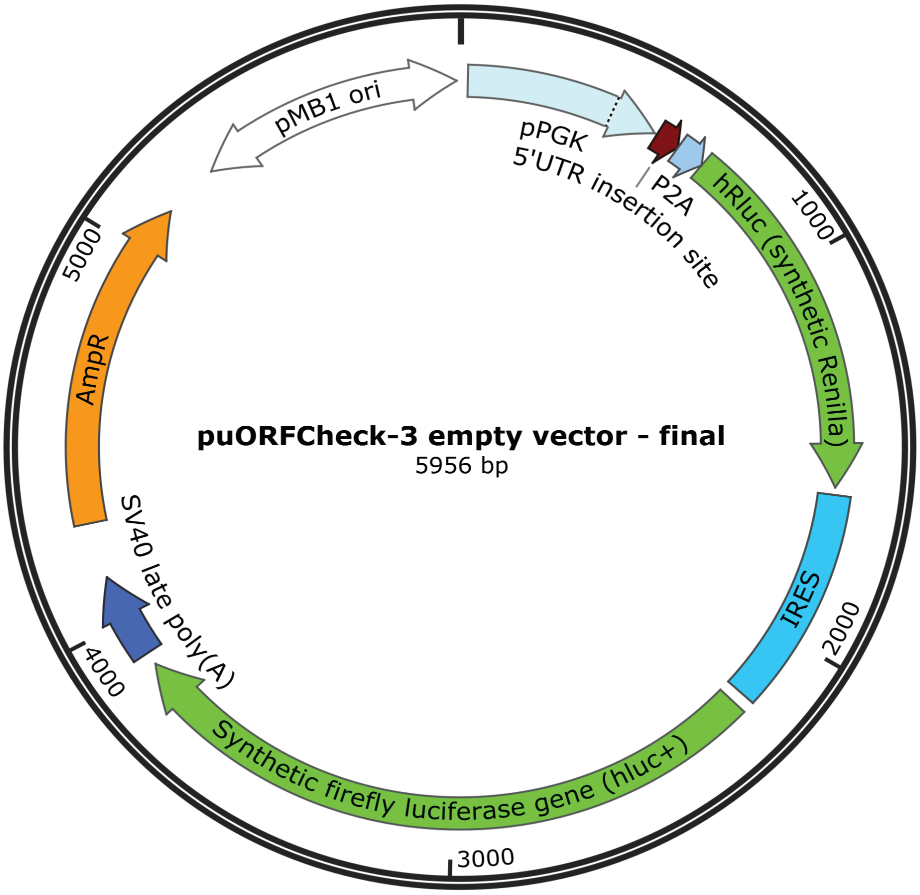

Supplementary Figure 6. puORF-Check3 plasmid map for dual-luciferase reporter assays. The plasmid puORF-Check3 is a 5.9 kb vector designed for mammalian expression of a bicistronic dual-luciferase system. It features a pMB1 origin of replication (pMB1 ori) to ensure efficient plasmid replication in *Escherichia coli* (*E. coli*). A phosphoglycerate kinase promoter (pPGK), located upstream of the NdeI restriction enzyme cloning site (designated as the $5'$ untranslated region ($5'$ UTR) insertion site), drives high-level expression in mammalian cells. A P2A self-cleaving peptide (P2A) is fused to the Renilla luciferase (*hRluc*) sequence to ensure that the $5'$ UTR reading frame is preserved. The plasmid encodes two luciferase genes, *hRluc* (Renilla luciferase) and *hluc*+ (firefly luciferase), transcribed together as a single mRNA transcript. These genes are separated by a viral internal ribosomal entry site (IRES), enabling simultaneous measurement of luminescence from both luciferase proteins in cell lysates. An ampicillin resistance gene (AmpR) is included to facilitate antibiotic selection in *E. coli* and propagation of successfully transformed cells. An SV40 late polyadenylation signal (SV40 late poly(A)) ensures proper transcript termination and polyadenylation.

Supplementary Data

Supplementary Data 1: ClinVar rG4-breaking variant annotations.

This dataset contains the transcript-level ΔG4mer scores for UTR variants in ClinVar annotated as pathogenic or benign. Only variants located within high-confidence rG4-forming regions (G4mer score > 0.7) are included. For each variant, the transcript with the most disruptive effect (i.e., minimum ΔG4mer) is retained. The file includes genomic coordinates, transcript IDs, G4mer scores, ΔG4mer values, and ClinVar annotations.

Supplementary Tables

The supplementary tables present a comprehensive list of rG4-altering variants from PMBB in breast cancer-associated genes. Additionally, they provide detailed information on the experimental sequences used in the dual-luciferase and CD experiments for functional and structural validation of rG4 formation.

Supplementary Table 1: rG4-altering variants
List of PMBB rG4-altering variants in breast cancer-associated genes.

Supplementary Table 2: Dual-luciferase experiment
Wild-type and mutant sequences used in dual-luciferase experiments with their respective predicted rG4 effects.

Supplementary Table 3: Circular dichroism experiment
Wild-type and mutant sequences used in CD experiments with their respective G4mer scores.

Supplementary Table 4: Counts of G4RNA sequences per experimental protocol
Number of sequences used in each of the experimental protocols included in the G4RNA dataset. G4RNA is a curated database of RNA sequences experimentally validated for rG4 formation and published across diverse studies and journals. After preprocessing to remove duplicates, conflicting annotations, and overlaps with the G4mer training set, we retained a total of n = 795 sequences spanning lengths from 14 to 1368 nucleotides. These sequences represent experimental validation across 24 distinct protocols (e.g., circular dichroism, in-line probing, SHALiPE, NMR), providing a heterogeneous benchmark for assessing model performance.

Supplementary Table 1. List of PMBB rG4-altering variants in breast cancer–associated genes. Chromosome:position:reference:alternate format is shown for each variant. An en dash (—) indicates no variant of that type was identified for the gene.

Gene	rG4-disrupting variant IDs	rG4-forming variant IDs
BRCA1	—	—
BRCA2	—	—
ATM	11:108315883:G:A, 11:108267235:G:A, 11:108267250:T:C	11:108249043:C:G, 11:108267283:A:G, 11:108267256:A:G, 11:108267281:C:T
CHEK2	22:28725338:T:C, 22:28734725:G:A, 22:28699929:C:G	22:28742305:T:C
PALB2	—	—
BARD1	—	2:214745828:T:C
RAD51C	—	17:58692618:C:T, 17:58692631:A:G
RAD51D	17:35119690:C:T, 17:35103500:C:T	17:35119674:G:A, 17:35103455:T:C, 17:35103492:G:A
TP53	—	17:7670704:A:C
PTEN	—	—
MSH2	—	—
PMS2	7:6009072:C:A, 7:6009094:A:G, 7:6009037:C:T, 7:6009072:C:T, 7:6009026:A:G, 7:5987298:C:T, 7:5987289:C:T	7:6009008:A:C, 7:5987277:G:A, 7:5987311:G:A
MSH6	2:47784238:G:C, 2:47799510:G:A, 2:47799508:G:C, 2:47799509:T:C	2:47783185:T:G, 2:47784095:C:A, 2:47790942:A:G, 2:47784209:C:G
MLH1	3:36993612:G:C, 3:36993568:T:C, 3:36993600:G:A	3:36993883:T:G, 3:36993556:C:G, 3:36993584:G:A, 3:36993616:A:G
CDH1	—	16:68828289:C:T, 16:68833528:C:A
CDKN2A	9:21971299:C:A, 9:21968600:T:C, 9:21968611:C:T, 9:21968615:C:T	9:21970998:G:C, 9:21970932:C:T, 9:21968585:G:A, 9:21968618:G:C

Supplementary Table 2. Sequences used in dual-luciferase reporter assays. WT, wild type; Mut, variant sequence from PMBB with the corresponding G4mer-predicted variant effect. Variant positions are highlighted in red.

Transcript	Construct	Sequence	Variant ID	Variant Effect	5'UTR length (nt)
EPN3-201	WT	GGAGTGCTGCCTGGCGCTGGCTAGGAGGCAAACGCACGCGG<br>GAAGAGCTGCTACCCATTCCAGGGACCCTGCCGCTGCCCCT<br>CTGAGGGGTCTGCACCTCCTGGGAGCAGGTGGGTCTCTGGG<br>ACGAGGGTCCATGGTGGATGGCTCTGGAGACGCTCCCGAGG<br>CTGTGCCGTCCCGCTGCTGCACAGGTCGGAGGGTCACCGCA<br>GAGGCTACTCGGGCTGGGGCTGGGGCCGAGGGAGCCCGCAC<br>TGGAGCCCCATGTGGAACCCAAGGATGCAGCTGCTCTGCTA<br>ACACGGCAGCCCATCCTTCAAGACTGTGACCTCGCCACAGT<br>GGCCCTCAGCCCTCCACCTCCGGCGGGGGCGAGGGCCACCC<br>ACCTCCAAGTCTCCAGCC	–	–	387
	Mut	GGAGTGCTGCCTGGCGCTGGCTAGGAGGCAAACGCACGCGG<br>GAAGAGCTGCTACCCATTCCAGGGACCCTGCCGCTGCCCCT<br>CTGAGGGGTCTGCACCTCCTGGGAGCAGGTGGGTCTCTGGG<br>ACGAGGGTCCATGTTGGATGGCTCTGGAGACGCTCCCGAGG<br>CTGTGCCGTCCCGCTGCTGCACAGGTCGGAGGGTCACCGCA<br>GAGGCTACTCGGGCTGGGGCTGGGGCCGAGGGAGCCCGCAC<br>TGGAGCCCCATGTGGAACCCAAGGATGCAGCTGCTCTGCTA<br>ACACGGCAGCCCATCCTTCAAGACTGTGACCTCGCCACAGT<br>GGCCCTCAGCCCTCCACCTCCGGCGGGGGCGAGGGCCACCC<br>ACCTCCAAGTCTCCAGCC	17:50532871:G:T	rG4-breaking	387
MSH6-203	WT	GAGTCCGGTGGTGTGGGGTGCGAAAGGAGGTTCCTCGGCCG<br>GCGCGGAGATAGTGAGTTGGGGCTCCAGTAGTCGATCGAGG<br>TAGACACTTAGAGGTAGTTAAGAGCCGCGGTCGCCGAGACG<br>CCTTGGGGACGGTGGGCCTTCGGCCTAGTTGTGACTTCTCA<br>CCAGGAGATTTGGTTTGGGCCAAG	–	–	188
	Mut	GAGTCCGGTGGTGTGGGGTGCGAAAGGAGGTTCCTCGGCCG<br>GCGCGGAGATAGTGAGTTGGGGCTCCAGTAGTCGATCGAGG<br>TAGACACTTAGAGGTAGTTAAGAGCCGCGGTCGCCGAGACG<br>CCTTGGGGACGGTGGGCCTTCGGCCTAGTTGTGACTTCTCA<br>CCGGGAGATTTGGTTTGGGCCAAG	2:47790942:A:G	rG4-forming	188

Supplementary Table 3. Circular dichroism experiment Wild-type and mutant sequences used in CD experiments with their respective G4mer scores.

Gene	Construct	Sequence	G4mer score
EPN3	WT	GGGACGAGGGTCCATGGTGGAT	0.915545
EPN3	Mut	GGGACGAGGGTCCATGTTGGAT	0.132675
MSH6	WT	CCAGGAGATTTGGTTTGGGC	0.409456
MSH6	Mut	CCGGGAGATTTGGTTTGGGC	0.773396

Supplementary Table 4. Counts of filtered G4RNA sequences per experimental protocol.

Experimental protocol	n
circular dichroism (CD)	180
in line probing	169
melting temperature (TM)	118
in cellulo luciferase assay (or other reporter gene expression)	141
in vitro luciferase assay (or other reporter gene expression)	27
reverse transcription termination	29
RNAse T1 probing	24
N-methyl mesoporphyrin IX binding	23
FMRP cation dependant binding	6
nuclear magnetic resonance	21
selective 2′-hydroxyl acylation analyzed by primer extension (SHAPE)	4
crystal x-ray scattering	1
electrophoresis mobility shift assay (EMSA) with BG4 antibody	1
native gel mobility	18
1-cyclohexyl-(2-morpholinoethyl)carbodiimide metho-p-toluene sulfonate probing (CMCT probing)	2
RNAse V1 probing	2
dimethylsulfate probing (DMS probing)	11
kethoxal probing	2
fluorescence spectroscopy	1
selective 2′-hydroxyl acylation analyzed by lithium ion based primer extension (SHALiPE)	6
RNAse T2 probing	1
dimethylsulfate probing analyzed by lithium ion based primer extension (DMSLiPE)	1
small angle x-ray scattering (SAXS)	4
electrophoresis mobility shift assay (EMSA) with DHX36 (RHAU)	3
**Total**	**795**
