# Supplementary material for: G4mer: An RNA language model for transcriptome-wide identification of G-quadruplexes and disease variants from population-scale genetic data
Source: Nat Commun. 2025 Nov 20;16:10221. doi: 10.1038/s41467-025-65020-7 (PMC12635080; doi:10.1038/s41467-025-65020-7)
Supplement: Supplementary file 2 — Description of Additional Supplementary Files [file 41467_2025_65020_MOESM2_ESM.pdf]

Description of Additional Supplementary Files

Supplementary Data 1: G4mer predictions for ClinVar UTR variants List of all ClinVar-annotated 5’ and 3’ UTR single-nucleotide variants (benign and pathogenic) that fall within high-confidence rG4 windows (wild-type G4mer score > 0.7). For each variant, we report genomic coordinates, reference/alternate alleles, transcript ID, wild-type and mutant G4mer scores, and ΔG4mer.
