# Supplementary material for: G4mer: An RNA language model for transcriptome-wide identification of G-quadruplexes and disease variants from population-scale genetic data
Source: Nat Commun. 2025 Nov 20;16:10221. doi: 10.1038/s41467-025-65020-7 (PMC12635080; doi:10.1038/s41467-025-65020-7)
Supplement: Supplementary file 4 — Reporting Summary [file 41467_2025_65020_MOESM4_ESM.pdf]

nature portfolio

Corresponding author(s): Yoseph Barash

Last updated by author(s): Aug 7, 2025

Reporting Summary

Nature Portfolio wishes to improve the reproducibility of the work that we publish. This form provides structure for consistency and transparency in reporting. For further information on Nature Portfolio policies, see our Editorial Policies and the Editorial Policy Checklist.

Statistics

For all statistical analyses, confirm that the following items are present in the figure legend, table legend, main text, or Methods section.

n/a	Confirmed	
☐	☒	The exact sample size (*n*) for each experimental group/condition, given as a discrete number and unit of measurement
☐	☒	A statement on whether measurements were taken from distinct samples or whether the same sample was measured repeatedly
☐	☒	The statistical test(s) used AND whether they are one- or two-sided<br>*Only common tests should be described solely by name; describe more complex techniques in the Methods section.*
☐	☒	A description of all covariates tested
☐	☒	A description of any assumptions or corrections, such as tests of normality and adjustment for multiple comparisons
☐	☒	A full description of the statistical parameters including central tendency (e.g. means) or other basic estimates (e.g. regression coefficient) AND variation (e.g. standard deviation) or associated estimates of uncertainty (e.g. confidence intervals)
☐	☒	For null hypothesis testing, the test statistic (e.g. *F*, *t*, *r*) with confidence intervals, effect sizes, degrees of freedom and *P* value noted<br>*Give P values as exact values whenever suitable.*
☒	☐	For Bayesian analysis, information on the choice of priors and Markov chain Monte Carlo settings
☒	☐	For hierarchical and complex designs, identification of the appropriate level for tests and full reporting of outcomes
☐	☒	Estimates of effect sizes (e.g. Cohen's *d*, Pearson's *r*), indicating how they were calculated

Our web collection on statistics for biologists contains articles on many of the points above.

Software and code

Policy information about availability of computer code

Data collection

No software was used to collect data.

Data analysis

Custom Python and R scripts were written for analyses included in the paper

https://bitbucket.org/biociphers/g4mer/src

The dependencies are:

absl-py==2.0.0
accelerate==0.31.0
aiodns==2.0.0
aiofiles==0.8.0
aiohttp==3.9.3
aiosignal==1.3.1
anyio==4.0.0
appdirs==1.4.4
argcomplete==3.3.0
argh==0.31.2
argon2-cffi==23.1.0
argon2-cffi-bindings==21.2.0
arrow==1.3.0

```
astroid==2.15.8
asttokens==2.4.1
astunparse==1.6.3
async-lru==2.0.4
async-timeout==4.0.3
asyncio==3.4.3
attrs==23.1.0
avro==1.11.3
azure-common==1.1.28
azure-core==1.30.2
absl-py==2.0.0
accelerate==0.31.0
aiodns==2.0.0
aiofiles==0.8.0
aiohttp==3.9.3
aiosignal==1.3.1
anyio==4.0.0
appdirs==1.4.4
argcomplete==3.3.0
argh==0.31.2
argon2-cffi==23.1.0
argon2-cffi-bindings==21.2.0
arrow==1.3.0
astroid==2.15.8
asttokens==2.4.1
astunparse==1.6.3
async-lru==2.0.4
async-timeout==4.0.3
asyncio==3.4.3
attrs==23.1.0
avro==1.11.3
azure-common==1.1.28
azure-core==1.30.2
azure-identity==1.17.1
azure-mgmt-core==1.4.0
azure-mgmt-storage==20.1.0
azure-storage-blob==12.20.0
Babel==2.13.1
backoff==1.11.1
beautifulsoup4==4.12.2
biopython==1.81
bleach==6.1.0
blessed==1.20.0
bokeh==3.3.4
boto3==1.29.2
botocore==1.32.2
brokenaxes==0.6.1
cachetools==5.3.2
cattrs==23.2.3
cdislogging==1.1.1
certifi==2023.7.22
cffi==1.16.0
chanfig==0.0.100
charset-normalizer==3.3.0
click==8.1.7
coloredlogs==15.0.1
contourpy==1.2.0
cryptography==42.0.8
cycler==0.12.1
datacache==1.4.1
dataclasses-json==0.5.9
debugpy==1.8.0
decorator==4.4.2
defusedxml==0.7.1
Deprecated==1.2.14
dictionaryutils==3.4.10
dill==0.3.7
drsclient==0.2.3
et-xmlfile==1.1.0
exceptiongroup==1.1.3
executing==2.0.1
fastavro==1.8.4
fastjsonschema==2.19.0
filelock==3.12.4
flatbuffers==23.5.26
fonttools==4.44.3
```

```
fqdn==1.5.1
frozenlist==1.4.1
fsspec==2023.9.2
future==1.0.0
gast==0.5.4
gen3==4.22.4
gen3Dictionary==2.0.3
gen3users==1.0.3
gff3-parser==0.0.5
gffutils==0.13
gitdb==4.0.11
GitPython==3.1.43
google-api-core==2.18.0
google-auth==2.29.0
google-auth-oauthlib==0.8.0
google-cloud-core==2.4.1
google-cloud-storage==2.16.0
google-crc32c==1.5.0
google-pasta==0.2.0
google-resumable-media==2.7.0
googleapis-common-protos==1.63.0
gpustat==1.1.1
GPUtil==1.4.0
grpcio==1.59.2
gtfparse==2.5.0
h11==0.14.0
h5py==3.10.0
hail==0.2.131
httpcore==1.0.4
httpx==0.27.0
huggingface-hub==0.17.3
humanfriendly==10.0
humanize==1.1.0
idna==3.4
importlib-metadata==6.8.0
importlib-resources==6.1.1
indexclient==2.2.1
intervaltree==3.1.0
ipykernel==6.26.0
ipython==8.17.2
ipywidgets==8.1.1
isodate==0.6.1
isoduration==20.11.0
isort==5.13.2
janus==1.0.0
jedi==0.19.1
Jinja2==3.1.2
jmespath==1.0.1
joblib==1.3.2
jproperties==2.1.1
json5==0.9.14
jsonpointer==2.4
jsonschema==4.21.1
jsonschema-specifications==2023.11.1
jupyter==1.0.0
jupyter-console==6.6.3
jupyter-events==0.9.0
jupyter-lsp==2.2.0
jupyter_client==8.6.0
jupyter_core==5.5.0
jupyter_server==2.10.1
jupyter_server_terminals==0.4.4
jupyterlab==4.1.5
jupyterlab-pygments==0.2.2
jupyterlab-widgets==3.0.9
jupyterlab_server==2.25.1
keras==2.15.0
kiwisolver==1.4.5
lazy-imports==0.3.1
lazy-object-proxy==1.10.0
libclang==16.0.6
Markdown==3.5.1
MarkupSafe==2.1.3
marshmallow==3.21.1
marshmallow-enum==1.5.1
matplotlib==3.8.1
```

```
matplotlib-inline==0.1.6
matplotlib-venn==0.11.10
mccabe==0.7.0
memoized-property==1.0.3
mistune==3.0.2
ml-dtypes==0.2.0
mlxtend==0.23.1
mock==5.1.0
more-itertools==10.2.0
mpmath==1.3.0
msal==1.29.0
msal-extensions==1.2.0
msrest==0.7.1
multidict==6.0.5
multimolecule==0.0.4
mypy-extensions==1.0.0
natsort==8.4.0
nbclient==0.9.0
nbconvert==7.11.0
nbformat==5.9.2
ncls==0.0.68
nest-asyncio==1.5.8
networkx==3.2.1
notebook==7.0.6
notebook_shim==0.2.3
numpy==1.26.0
nvidia-cublas-cu12==12.1.3.1
nvidia-cuda-cupti-cu12==12.1.105
nvidia-cuda-nvrtc-cu12==12.1.105
nvidia-cuda-runtime-cu12==12.1.105
nvidia-cudnn-cu12==8.9.2.26
nvidia-cufft-cu12==11.0.2.54
nvidia-curand-cu12==10.3.2.106
nvidia-cusolver-cu12==11.4.5.107
nvidia-cusparse-cu12==12.1.0.106
nvidia-ml-py==12.555.43
nvidia-nccl-cu12==2.18.1
nvidia-nvjitlink-cu12==12.3.101
nvidia-nvtx-cu12==12.1.105
oauthlib==3.2.2
openpyxl==3.1.2
opt-einsum==3.3.0
orjson==3.10.5
overrides==7.4.0
packaging==23.2
pandarallel==1.6.5
pandas==2.1.3
pandocfilters==1.5.0
parsimonious==0.10.0
parso==0.8.3
patsy==0.5.6
pexpect==4.8.0
Pillow==10.1.0
platformdirs==4.0.0
plotly==5.22.0
polars==0.20.31
portalocker==2.10.0
progressbar33==2.4
prometheus-client==0.18.0
prompt-toolkit==3.0.41
proto-plus==1.23.0
protobuf==3.20.2
psutil==5.9.6
ptyprocess==0.7.0
pure-eval==0.2.2
py4j==0.10.9.7
pyarrow==14.0.2
pyasn1==0.5.0
pyasn1-modules==0.3.0
pybedtools==0.10.0
pybiomart==0.2.0
pycares==4.4.0
pycparser==2.21
pyensembl==2.3.13
pyfaidx==0.8.1.1
Pygments==2.16.1
```

```
PyJWT==2.8.0
pylint==2.17.7
pyparsing==3.1.1
pypfb==0.5.29
pyranges==0.0.129
pyrsistent==0.20.0
pysam==0.22.1
pyspark==3.5.1
python-dateutil==2.8.2
python-json-logger==2.0.7
pytz==2023.3.post1
PyVCF==0.6.8
PyYAML==6.0.1
pyzmq==25.1.1
qtconsole==5.5.1
QtPy==2.4.1
referencing==0.31.0
regex==2023.10.3
requests==2.31.0
requests-cache==1.2.1
requests-oauthlib==1.3.1
rfc3339-validator==0.1.4
rfc3986==1.5.0
rfc3986-validator==0.1.1
rich==12.6.0
rpds-py==0.13.0
rpy2==3.5.15
rsa==4.9
s3transfer==0.7.0
sacremoses==0.1.1
safetensors==0.4.0
scikit-learn==1.3.2
scipy==1.11.4
seaborn==0.13.0
Send2Trash==1.8.2
sentencepiece==0.1.99
serializable==0.4.1
shellingham==1.5.4
simplejson==3.19.2
six==1.16.0
smmap==5.0.1
sniffio==1.3.0
sorted-nearest==0.0.39
sortedcontainers==2.4.0
soupsieve==2.5
stack-data==0.6.3
statsmodels==0.14.1
StrEnum==0.4.15
sympy==1.12
tabulate==0.9.0
tenacity==8.4.1
tensorboard==2.15.1
tensorboard-data-server==0.7.2
tensorflow==2.15.0
tensorflow-estimator==2.15.0
tensorflow-io-gcs-filesystem==0.34.0
termcolor==2.3.0
terminado==0.18.0
threadpoolctl==3.2.0
tinycss2==1.2.1
tinytimer==0.0.0
tokenizers==0.11.1
tomli==2.0.1
tomlkit==0.12.5
torch==2.1.1
torchaudio==2.1.1
torchvision==0.16.1
tornado==6.3.3
tqdm==4.66.1
traitlets==5.13.0
transformers==4.29.0
triton==2.1.0
typechecks==0.1.0
typer==0.12.3
types-python-dateutil==2.8.19.14
typing-inspect==0.9.0
```

typing_extensions==4.8.0
tzdata==2023.3
tzlocal==5.2
uri-template==1.3.0
url-normalize==1.4.3
urllib3==1.26.19
uvloop==0.19.0
wcwidth==0.2.10
webcolors==1.13
webencodings==0.5.1
websocket-client==1.6.4
Werkzeug==3.0.1
wget==3.2
widgetsnbextension==4.0.9
wrapt==1.14.1
xlrd==2.0.1
xmltodict==0.13.0
xyzservices==2024.6.0
yarl==1.9.4
zipp==3.17.0

For manuscripts utilizing custom algorithms or software that are central to the research but not yet described in published literature, software must be made available to editors and reviewers. We strongly encourage code deposition in a community repository (e.g. GitHub). See the Nature Portfolio guidelines for submitting code & software for further information.

Data

Policy information about availability of data

All manuscripts must include a data availability statement. This statement should provide the following information, where applicable:

- Accession codes, unique identifiers, or web links for publicly available datasets
- A description of any restrictions on data availability
- For clinical datasets or third party data, please ensure that the statement adheres to our policy

Transcript, annotation files, and genome files used to extract UTR sequences are available on GENCODE https://www.gencodegenes.org/human/release_29.html. rG4-seeker data is available as a supplementary material of Chow et al (2020)31. G4RNA is accessible through http://scottgroup.med.usherbrooke.ca/G4RNA/. gnomAD v3.1.2 variants and their corresponding alternate allele frequency and CADD scores, as well as the genomic constraint dataset can be publicly accessed at https://gnomad.broadinstitute.org/downloads#. The phenotype and genotype datasets from Penn Medicine BioBank analyzed are available via the data access process https://pmbb.med.upenn.edu/investigators.php. Sequences used in dual luciferase experiments are available in Supplementary Table 2. Sequences used in circular dichroism experiments are available in Supplementary Table 3.

Research involving human participants, their data, or biological material

Policy information about studies with human participants or human data. See also policy information about sex, gender (identity/presentation), and sexual orientation and race, ethnicity and racism.

Reporting on sex and gender	Sex of origin for cell lines used: HuH-7 – Male (human); CHO – Female (hamster ovary); NIH3T3 – Mouse (embryo)
Reporting on race, ethnicity, or other socially relevant groupings	Not applicable
Population characteristics	Not applicable
Recruitment	Not applicable
Ethics oversight	Not applicable

Note that full information on the approval of the study protocol must also be provided in the manuscript.

Field-specific reporting

Please select the one below that is the best fit for your research. If you are not sure, read the appropriate sections before making your selection.

☒ Life sciences ☐ Behavioural & social sciences ☐ Ecological, evolutionary & environmental sciences

For a reference copy of the document with all sections, see nature.com/documents/nr-reporting-summary-flat.pdf

Life sciences study design

All studies must disclose on these points even when the disclosure is negative.

Sample size	All data derived from publicly available datasets included all available samples.<br>Other datasets:<br>- Penn Medicine BioBank: European (N=29,362) and African (N=10,217)<br>- Dual luciferase replicates: 3<br>- Circular dichroism replicates: 3<br><br>No sample size calculation was done; 3-5 replicates were chosen following the standards and consistent with prior published protocols.
Data exclusions	Data exclusions were applied based on predefined criteria for different datasets. For Penn Medicine BioBank dataset, we only kept samples which are 2nd-degree unrelated in each ancestry group, resulting in two groups of samples of European (N=29,362) and African (N=10,217) genetic ancestries. Furthermore, exclusion criteria applied to variants include singletons and<br>high missing call rates (exceeding 0.1). gnomAD variants were filtered to retain only those that passed all quality control (QC) filters, as indicated by having FILTER value of None. We further excluded variants located in low complexity regions, decoy<br>regions, and segmental duplications by checking for the presence of 'lcr', 'decoy', or 'segdup' in the INFO field. Finally, we only included those those with a total observed allele count of at least 80% of the maximum number of sequenced alleles.
Replication	Data and associated scripts to replicate all analyses are in the Bitbucket repository at https://bitbucket.org/biociphers/g4mer/src/main/
Randomization	Samples were allocated based on predefined disease or control labels in the publicly available datasets, and no new randomization was performed in this study.
Blinding	Blinding is not applicable because we only used datasets generated in other studies

Reporting for specific materials, systems and methods

We require information from authors about some types of materials, experimental systems and methods used in many studies. Here, indicate whether each material, system or method listed is relevant to your study. If you are not sure if a list item applies to your research, read the appropriate section before selecting a response.

Materials & experimental systems

n/a	Involved in the study	
☒	☐	Antibodies
☐	☒	Eukaryotic cell lines
☒	☐	Palaeontology and archaeology
☒	☐	Animals and other organisms
☒	☐	Clinical data
☒	☐	Dual use research of concern
☒	☐	Plants

Methods

n/a	Involved in the study	
☒	☐	ChIP-seq
☒	☐	Flow cytometry
☒	☐	MRI-based neuroimaging

Eukaryotic cell lines

Policy information about cell lines and Sex and Gender in Research

Cell line source(s)	CHO and NIH3T3 acquired from ATCC. HuH-7 acquired from JCRB.<br>Catalog number: HuH-7 (JCRB, Cat# JCRB0403), CHO (ATCC, Cat# CCL-61), NIH3T3 (ATCC, Cat# CRL-1658)<br>Sex of origin: HuH-7 – Male (human); CHO – Female (hamster ovary); NIH3T3 – Mouse (embryo)
Authentication	Huh-7 cells were authenticated in June 2017 by short tandem repeat (STR) profiling (GenePrint® 10 System, Promega) and matched the expected reference profile. CHO and NIH3T3 cell lines have not been recently authenticated.
Mycoplasma contamination	Not tested.
Commonly misidentified lines<br>(See ICLAC register)	We have downloaded version 13 of the misidentified lines list rom 26 April 2024 from https://iclac.org/databases/cross-contaminations/. We did not find HuH-7, CHO, and NIH3T3 to be in the list of known misidentified cell lines.

Plants

Seed stocks	Not applicable
Novel plant genotypes	Not applicable
Authentication	Not applicable
